# JAK/STAT: Why choose a classical or an alternative pathway when you can have both?

**DOI:** 10.1111/jcmm.17168

**Published:** 2022-03-03

**Authors:** Léna Puigdevall, Camille Michiels, Clara Stewardson, Laure Dumoutier

**Affiliations:** ^1^ Experimental Medicine Unit de Duve Institute Université catholique de Louvain Brussels Belgium

**Keywords:** cytokine signalling, Janus kinase, noncanonical, signal transducer and activator of transcription, tyrosine‐independent

## Abstract

A subset of cytokines triggers the JAK‐STAT pathway to exert various functions such as the induction of inflammation and immune responses. The receptors for these cytokines are dimers/trimers of transmembrane proteins devoid of intracellular kinase activity. Instead, they rely on Janus kinases (JAKs) for signal transduction. Classical JAK‐STAT signalling involves phosphorylation of cytokine receptors' intracellular tyrosines, which subsequently serve as docking sites for the recruitment and activation of STATs. However, there is evidence to show that several cytokine receptors also use a noncanonical, receptor tyrosine‐independent path to induce activation of STAT proteins. We identified two main alternative modes of STAT activation. The first involves an association between a tyrosine‐free region of the cytokine receptor and STATs, while the second seems to depend on a direct interaction between JAK and STAT proteins. We were able to identify the use of noncanonical mechanisms by almost a dozen cytokine receptors, suggesting they have some importance. These alternative pathways and the receptors that employ them are discussed in this review.

## 1 INTRODUCTION

**FIGURE 1:**
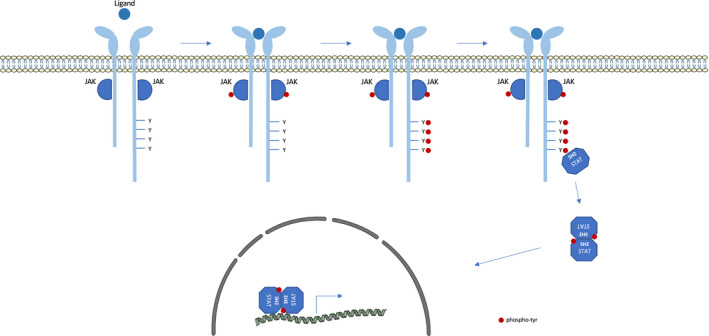
Canonical JAK‐STAT pathway. Schematic representation of the signalling cascade induced by cytokines that signal through the JAK‐STAT pathway. Once a cytokine binds to a specific receptor, it induces JAK transactivation. Activated JAKs then phosphorylate tyrosine residues in the intracellular domain of the receptor. These phosphotyrosines in turn serve as docking site for STAT recruitment, through their SH2 domain. Subsequently, JAKs phosphorylate tyrosine residue of STAT factors, resulting in STAT activation. The activated STATs form homo‐ and hetero‐dimers that migrate into the nucleus and regulate the transcription of specific genes

Processes such as cellular proliferation, differentiation, growth and apoptosis are regulated in part by numerous extracellular factors including cytokines. These glycoproteins, which have an immunomodulatory role, are also mainly secreted by cells of the immune system. They perform their paracrine, juxtacrine, autocrine or endocrine actions by binding to specific cytokine receptors at the cell surface. A subset of cytokines triggers the JAK/STAT pathway to exert biological functions^1^, ^2^ (Figure 1). Their receptors are homodimers or hetero‐dimers/trimers of transmembrane proteins that lack intracellular kinase activity. Instead, they rely on constitutive association with intracellular tyrosine kinases called Janus kinases (JAK) for the transduction of cytokine signalling.

The Janus kinase family consists of four members: JAK1, JAK2, JAK3 and TYK2 that are ubiquitously expressed, except for JAK3 whose expression is restricted to cells of the haematopoietic lineage. JAKs, which are associated with the juxta‐membrane domain of the receptor, become active when cytokine binds the receptor. Following this binding, the receptor undergoes intracellular conformational changes that result in its re‐orientation or oligomerization. This initiates the signalling process by juxtaposing the JAKs that are now able to trans‐phosphorylate each other. The primary targets of activated JAKs are specific tyrosine residues in the cytoplasmic tail of the receptor. Once phosphorylated, these tyrosines become docking sites for signalling proteins such as signal transducers and activators of transcription (STAT) that contain a Src homology 2 domain (SH2). Subsequently, JAKs phosphorylate tyrosine residues of STAT factors, which results in STAT activation.

Signal transducers and activators of transcription are transcription factors that reside in the cytoplasm prior to cytokine binding. The STAT family has seven members: STAT1, STAT‐2, STAT‐3, STAT‐4, STAT‐5a, STAT‐5b and STAT‐6. They contain six functional regions: an N‐terminal domain mediating STAT oligomerization, followed by a coiled‐coil domain, a DNA‐binding domain, a linker region, a SH2 domain and, finally, a carboxy‐terminal transactivation domain. This last domain contains a conserved tyrosine that can be phosphorylated by JAK, thereby allowing for parallel STAT homo‐ or heterodimerization via reciprocal SH2‐phosphotyrosine interactions. Antiparallel dimer conformation, in which the coiled‐coil domain of one monomer interacts with the DNA‐binding domain of the second monomer, is also observed. The activated STAT factors permanently alternate between both dimer conformations. Only the parallel STAT dimers migrate to the nucleus, thanks to importins, and bind to specific DNA sequences in the promoter regions to regulate the transcription of their respective target genes. After dissociation from DNA, STAT factors are dephosphorylated and can be exported from the nucleus. These cytoplasmic STAT proteins are then available for another cycle of phosphorylation and nucleocytoplasmic shuttling.

The activation of the JAK/STAT pathway is finely tuned and eventually down‐regulated in order to limit the action of downstream effectors in time and amplitude. Constitutive protein tyrosine phosphatases come into play to dephosphorylate tyrosine residues involved in activation and recruitment.^2^ Additionally, suppressors of cytokine signalling or SOCS, whose expression is induced following cytokine stimulation, regulate JAK/STAT activity by three different mechanisms: competing with STATs for receptor association, binding directly to JAKs and interfering with substrate recognition, and promoting ubiquitination and subsequent proteasomal degradation of JAKs.^3^, ^4^, ^5^ Moreover, protein inhibitor of activated STAT (PIAS) bind STAT dimers and also have multiple means of action, such as inhibiting DNA binding or recruiting transcriptional repressors.^4^

While signalling by cytokine receptors lacking kinase domains always starts with JAK, downstream kinases such as Src or the MAPKinases are also able to phosphorylate STATs. In this review, however, we will focus on the JAK/STAT pathway and more particularly, noncanonical ways of activating STATs. Indeed, while phosphotyrosines of cytokine receptors are believed to be major docking sites for STAT factors, the role of these residues should be nuanced, as STAT activation has been observed with receptors lacking tyrosines. In general, this alternative mode of STAT activation is not exclusive: canonical and noncanonical pathways collaborate.

Here, we focus on two alternative mechanisms of STAT activation that do not rely on receptor phosphotyrosines. The first depends on a region of the receptor that is devoid of tyrosines and, hence, is termed ‘receptor tyrosine independent’. The other is characterized by direct interaction between STAT and receptor‐associated JAKs and will be referred to as ‘JAK‐direct’.

## 2 RECEPTOR‐PHOSPHOTYROSINE‐INDEPENDENT MECHANISMS

**FIGURE 2:**
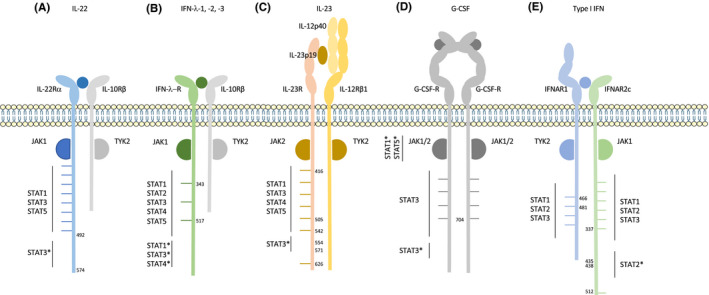
Receptors that signal through a phosphotyrosine‐independent pathway. (A) hIL‐22R, (B) hIFN‐λ‐R, (C) mIL‐23R, (D) hG‐CSFR and (E) hIFNAR signal through a classical JAK‐STAT signalling pathway as well as an alternative pathway. This alternative pathway is independent of tyrosines of the receptor. Graphs are not to scale

Usually, STATs are preferentially recruited to particular amino acid motifs surrounding phosphotyrosines: pYxxP for STAT1, pYxxQ for STAT3, pYxxL for STAT5 and pYxxF for STAT6.^6^ Some flexibility in binding site is, however, observed. For example, in the IL‐9R, which is associated with the γc chain, one tyrosine alone is responsible for the entire signalling process. Indeed, mutation of Y407 (located in the YLPQ site) is sufficient to abolish STAT1, STAT3 and STAT5 phosphorylation.^7^ Although phosphotyrosine importance is well established, several receptors activate STAT factors through a region of the receptor that does not contain a classical phosphotyrosine binding site for STAT. Figure 2 describes the receptors that signal through such a tyrosine‐independent mechanism.

### 2.1 IL‐22Rα

The receptor of IL‐22 is composed of two subunits: IL‐22Rα, responsible for signalling, and IL‐10Rβ (Figure 2A). Once bound to its receptor, IL‐22 triggers the activation of STAT3 and, to a lesser extent, of STAT1 and STAT5. Surprisingly, a tyrosine‐less receptor, lacking all 8 cytoplasmic tyrosines of IL‐22Rα, is still able to activate STAT3. This demonstrates that tyrosines are not absolutely required to activate STAT3, in contrast to what is observed for STAT1 and STAT5. Using truncated forms of the receptor in BW5147 T lymphoma cells, we were able to demonstrate that the C‐terminal (C‐ter) part of the receptor (aa 492–574) plays an important role in this noncanonical pathway although it does not contain tyrosines. Importantly, the activation of STAT3 is maximal when IL‐22Rα contains both its tyrosines and its C‐terminal part, demonstrating that canonical and noncanonical mechanisms cooperate to strongly activate STAT3.^8^

**FIGURE 3:**
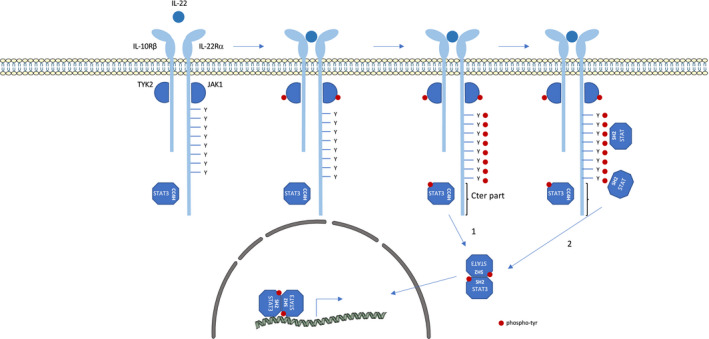
Cooperation of canonical and noncanonical activation pathways of IL‐22R. Schematic representation of the signalling cascade induced by IL‐22. The C‐terminal part of IL‐22R is pre‐associated with the coiled‐coil domain of STAT3. IL‐22 binds to IL‐22R leading to JAK transactivation. 1) Noncanonical STAT3 activation: Activated JAKs phosphorylate tyrosine residues on pre‐associated STAT3 leading to its activation, independently of receptor tyrosine residues. 2) Canonical STAT activation: Activated JAKs also phosphorylate tyrosine residues in the intracellular domain of the receptor. These phosphotyrosines serve as docking sites for STAT recruitment through their SH2 domains. Activated STATs form homo‐ and hetero‐dimers that migrate into the nucleus and regulate the transcription of specific genes.

The alternative mechanism requires a pre‐association of STAT3 to the receptor. This pre‐association step does not require STAT3's SH2 domains. Indeed, GST pull‐down experiments showed that STAT3, but not STAT1 or STAT5, is constitutively associated via its coiled‐coil domain with the C‐terminal part of the IL‐22Rα. Moreover, the coiled‐coil domain of STAT3 seems to be sufficient for this activation (Figure 3): Wild‐type STAT5 is not phosphorylated by a tyrosine‐less receptor; however, replacement of its coiled‐coil domain by that of STAT3 renders it phosphorylable despite the absence of receptor tyrosines.^8^ This was recently corroborated in a study in which a monobody targeting the coiled‐coil domain of STAT3 interrupts its binding to the IL‐22Rα C‐terminal part.^9^ This alternative mechanism is observed in cells overexpressing mutant forms of the receptor, and also when the receptor is expressed at physiological levels. Using a CRISPR/Cas9 approach, we confirmed that IL‐22Rα C‐ter‐dependent activation of STAT3 occurred in cells with endogenous expression of IL‐22Rα. In addition, in a psoriasis‐like dermatitis model induced by imiquimod in which IL‐22 and STAT3 are known to be deleterious, mice expressing a C‐terminally truncated version of IL‐22Rα (ΔCter^mut/mut^ mice) are protected from the development of skin lesions, demonstrating the importance of the noncanonical STAT3 pathway. In this model, ΔCter^mut/mut^ mice show less thickening of ear epidermis as compared to WT littermate mice.^10^

### 2.2 IFN‐λ‐R1

Alternative activation of STAT is similarly observed for the IFN‐λ‐R, which also uses the IL‐10Rβ accessory subunit (Figure 2B). This receptor complex mediates the activation of STAT1, STAT2, STAT3, STAT4 and STAT5 in response to type III interferons: IFN‐λ1, IFN‐λ2 and IFN‐λ3 (also called IL‐29, IL‐28A and IL‐28B). With a tyrosine‐less IFN‐λ‐R1, the phosphorylation of STAT1, STAT3 and STAT4 is still detected in response to IFN‐λ1.^11^ Here again, by using truncated forms of the receptor, we were able to show that the C‐terminal part of the IFN‐λ‐R1 is required for the noncanonical pathway involved in STAT1, STAT3 and STAT4 activation (unpublished data from the laboratory). In contrast, STAT2 and STAT5 phosphorylation, responsible for the anti‐proliferative and antiviral effects of IFN‐λ1, depends solely on two of the three tyrosine residues contained in the receptor's cytoplasmic domains (Tyr343 and Tyr517).^11^

### 2.3 IL‐23R

The IL‐23 cytokine signals through a heterodimeric receptor composed of the IL‐23R and the shared IL‐12Rβ1 (Figure 2C). It mainly activates STAT3, although other pathways such as STAT1, STAT4, STAT5, MAPK and Pi3K/Akt are also induced.^12^ The intracellular part of the murine IL‐23R contains seven tyrosines, four of which (Tyr416, Tyr504, Tyr542 and Tyr626) are involved in STAT3 activation in Ba/F3 cells transfected with IL‐23 receptors. Mutation of these four tyrosines into phenylalanines leads to a partial decrease of STAT3 activation. In addition to the classical activation of STAT3, a phosphotyrosine‐independent STAT3 activation motif was identified in IL‐23R. Indeed, a deletion of 17 amino acids (aa 554–571) in the IL‐23R with the four tyrosine‐to‐phenylalanine mutations completely blocks STAT3 activation. Constitutive association of STAT3/IL‐23R could not be detected by co‐immunoprecipitation experiments, suggesting that an intermediate protein may be involved.^13^

### 2.4 G‐CSF‐R

Granulocyte colony‐stimulating factor (G‐CSF) plays a crucial role in granulopoiesis. In the presence of G‐CSF, 2 chains of G‐CSF‐R form a homodimer that induces the activation of STAT1, STAT3 and STAT5 (Figure 2D). It was shown that cells expressing a full‐length G‐CSF‐R do not require any of its 4 tyrosines for STAT3 signalling.^14^, ^15^ In fact, Ba/F3 cells expressing a receptor in which all tyrosines are mutated into phenylalanines still present an activation of STAT3 that seems to depend on the C‐terminal part of the receptor.^15^, ^16^ When the C‐terminus region is truncated, STAT3 activation is enabled by tyrosine 704 (Tyr704), which seems to be the major docking site for STAT3 in this context. These results demonstrate that both the C‐ter part of the receptor and Tyr704 are involved in strong STAT3 activation. Functional studies in murine myeloid cell lines showed that the C‐terminal region of G‐CSFRs is required for G‐CSF‐induced neutrophil differentiation and maturation.^17^, ^18^, ^19^ Truncation of this region is observed in vivo in individuals with severe congenital neutropenia (SCN) or acute myeloid leukaemia (AML). Similarly, *Csf3r*‐Δ715 mice expressing a truncated form of the receptor (conserving Tyr704) also show basal neutropenia. Given these results, loss of the C‐terminal part of the receptor, which is responsible for a part of STAT3 activation, might explain the basal neutropenia seen in *Csf3r*‐Δ715 and in SCN patients.^15^ However, besides STAT3 modification, truncated G‐CSFR also has a prolonged half‐life at the plasma membrane. This results in an elevated G‐CSF‐induced proliferative response and sustained activation of STAT5 in myeloid progenitors, which could promote the clonal expansion of haematopoietic stem cells and progenitor cells.^20^ Of note, mutation of GCSFR seems to be a second hit following mutation of Elane (encoding neutrophil elastase) observed in the vast majority of SCN patients, and leading to neutropenia.^19^ These patients receive pharmacologic doses of GCSF to increase neutrophil numbers. This treatment would then select for truncation of GCSFR, which then promotes acute myeloid leukaemia.

It is also worth noting that at saturating G‐CSF concentrations, activation of STAT3 is mediated by the C‐terminus part of the receptor in a non‐tyrosine‐dependent manner. In contrast, at a low ligand concentration, it is mainly the tyrosine residues, particularly Tyr704 and 744, that support STAT3 activation. Physiologically, the effects mediated by G‐CSF on basal granulopoiesis occur at low ligand concentrations and are therefore mainly dependent on tyrosine residues. However, during bacterial infections, G‐CSF treatments or haematopoietic recovery after myelosuppressive treatment, levels of G‐CSF rise. In these conditions, STAT3 activation is likely to be mainly dependent on the C‐ter of the receptor. This means that two distinct modes of STAT3 activation by G‐CSF have been highlighted. Nonetheless, the tyrosine‐dependent and tyrosine‐independent routes of activation are functionally equivalent, and both are JAK2‐dependent.^15^ However, no SH2‐independent interaction has been detected between STAT3 and G‐CSF‐R‐C‐ter by in vitro binding assay or co‐immunoprecipitation.^15^ An intermediate factor could provide a phosphotyrosine docking site for STAT3, but no such molecule has yet been discovered.^15^

An alternative mechanism of activation of STAT1 and STAT5 was also described for G‐CSF signalling. This seems to rely on a JAK‐direct interaction and will therefore be discussed in the second part of this review.

### 2.5 IFNAR

Cellular responses to type I interferons (IFN‐β, IFN‐ε and IFN‐ω and 13 subtypes of IFN‐α) require interaction with their receptor, which is composed of two subunits, IFNAR1 and IFNAR2c (Figure 2E). Type I interferons preferentially activate STAT1 and STAT2, but STAT3 can also be phosphorylated in response to these cytokines.

Multiple docking sites have been described for STAT2 on the human IFNAR. Two require phosphotyrosine residues, either on the hIFNAR1 (Tyr466 and Tyr481) or hIFNAR2c chains (Tyr337 and Tyr512). Of note, the corresponding tyrosines in mouse IFNAR2c: Tyr510 and Tyr335, play a critical role in IFN‐I response in fibroblasts and macrophages. This includes activation of STAT2 and also STAT‐1 and STAT‐3, as well as the expression of target genes and antiviral activity.^21^

A third docking site, located on the IFNAR2c chain, is devoid of tyrosines.^22^, ^23^, ^24^, ^25^ A pre‐association was observed between this chain and the N‐terminal domain of STAT2. Indeed, STAT2 co‐precipitates with resting IFNAR2c and this association is increased after IFN treatment.^22^, ^23^, ^24^, ^26^, ^27^, ^28^ Many studies have attempted to define the region of IFNAR2c required for this interaction. GST pull‐down experiments have shown that amino acids 418–462 of the receptor are involved in the association, more precisely the acidic block DDED (435–438).^28^ Binding of STAT2 depends on its N‐terminal domain (including the coiled‐coil domain), since a STAT1 chimera containing the N‐terminal domain of STAT2 is able to act as a STAT2‐like molecule and bind IFNAR2c.^22^, ^27^ Nguyen et al^28^ showed that the coiled‐coil domain is not sufficient and that the SH2 domain of STAT2 could play a role in its interaction with the acidic portion of the receptor. Of note, glutamate residues are involved in the interaction between the receptor and the JAK factors. It was shown that the SH2 domains of JAKs interact with a motif containing glutamate and hydrophobic residues in the BOX2 of the cytokine receptor.^29^, ^30^ This may also be the case for the interaction between the SH2 of STAT2 and the DDED motif. While the binding sites of STAT2 on IFNAR1 and IFNAR2c are well described, data on the importance of these binding sites in STAT2 activation are conflicting. Some papers describe the tyrosine‐dependent and tyrosine‐independent binding sites on IFNAR2c to be equally involved in STAT2 activation.^23^, ^24^ Others on the contrary claim that only the IFNAR2c tyrosines are required and that the constitutive binding site is dispensable.^25^, ^28^ These discrepancies might be explained by the models used, with the truncation^23^, ^24^ versus point mutation of the receptor^28^ leading to different conformational modifications. It is, however, clear that in some conditions, STAT2 activation is unconventional and depends on a region lacking tyrosine residues (aa 418–462).

Many studies show that STAT1 activation relies on STAT2 phosphorylation, suggesting that phosphorylated‐STAT2 is used as a docking site by STAT1 or even that STAT1 is pre‐associated with the IFNAR2c via STAT2.^22^, ^27^, ^31^ Only one study describes an additional binding site for STAT1, on Tyr466 of IFNAR1.^22^

Concerning STAT3, two studies show conflicting results: Nadeau et al^23^ describe STAT3 activation as tyrosine‐independent while others show a lack of STAT3 activation in the absence of IFNAR2c tyrosines.^32^ Both studies were performed on human IFNAR2c in which all tyrosine residues were mutated into phenylalanines, but used different cellular models, either L929 cells, a mouse fibrosarcoma, or IFNAR2c‐deficient U5A cells, a mutant version of HT 1080 human sarcoma cell line. Differences in the JAK/STAT pathway between species have been documented and might thus explain these conflicting results.^21^

To summarize, STAT2 can be activated by both classical and alternative means while STAT1 phosphorylation relies on STAT2 phosphorylation. STAT3 activation also seems to take place in a tyrosine‐independent manner, at least in some conditions.

### 2.6 Conclusions

**FIGURE 4:**
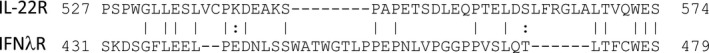
Alignment of IL‐22Rα and IFN‐λ‐R. Alignment of C‐terminal amino acids (527–574) of IL‐22Rα and (395–449) of IFN‐λ‐R. 16 residues are shared between these motifs. Pairwise alignment by ClustalW

Several receptors seem to activate STAT factors using a receptor tyrosine‐independent mechanism. To date, it is difficult to define whether this is a common mechanism. One striking observation is that STAT3 is often involved in alternative activation. This is notably the case for IL‐22Rα, IFN‐λ‐R1, G‐CSF‐R and IL‐23R. Alignment of the C‐terminal regions of these receptors does not identify a consensus motif, suggesting that different mechanisms are involved. However, when we aligned them in pairs, we found a consensus motif between IL‐22Rα and IFN‐λ‐R that could explain their unconventional binding with STAT3 (Figure 4). Further studies are required to define whether STAT3 is also pre‐associated with IFN‐λ‐R1, whether the common residues are involved in the noncanonical STAT3 activation induced by these two receptors and whether other receptors could also use this alternative mechanism. It might also be important to define whether pre‐association competes with the role of unphosphorylated STATs that constantly shuttle into the nucleus where they regulate gene transcription, promote heterochromatin stability and suppress tumour progression.^33^, ^34^

It is worth mentioning that mechanisms used to induce noncanonical activations of STATs are probably mediated by JAK. Although there is no evidence of this for IL‐23R, IFNAR or IFNλR, studies on other receptors have demonstrated the importance of JAKs. The alternative activation of STAT3 by G‐CSF‐R seems to require functional JAK2 since the use of the JAK2 inhibitor AG‐490 completely abrogates STAT3 activation.^15^ We obtained similar results with the tyrosine‐less IL‐22Rα: In the presence of the CMP6, a pan‐JAK inhibitor, STAT3 phosphorylation is in fact completely abolished (unpublished data).

## 3 JAK‐DIRECT MECHANISMS

**FIGURE 5:**
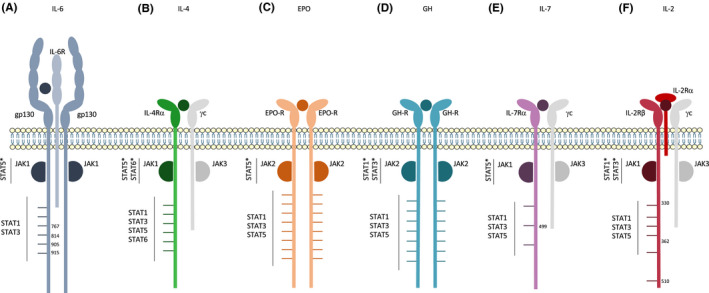
Receptors that signal through a JAK‐Direct pathway (A) hIL‐6R, (B) hIL‐4R, (C) hEPO‐R, (D) hGH‐R, (E) hIL‐7R and (F) hIL‐2R signal through a classical JAK‐STAT signalling pathway as well as an alternative pathway that is dependent on a JAK‐direct interaction between STAT proteins and JAKs. Graphs are not to scale

Janus kinase proteins interact through their SH2 and FERM domains with the proximal region of cytokine receptors containing box motifs: box 1, a proline‐rich region, and box 2, a region rich in hydrophobic residues and a glutamate. These are conserved between cytokine receptors and are known to be crucial for their interaction with JAK kinases.^6^ Some studies have been able to demonstrate a noncanonical direct interaction between JAK and STAT proteins. Indeed, the interaction between the JH2 (pseudokinase) domain of JAK1, JAK2, JAK3 and the carboxy‐terminal region of STAT5, but not STAT1 or STAT3, was demonstrated using the yeast two‐hybrid system, co‐immunoprecipitation and GST pull down.^35^, ^36^ Thus, studies showing that the proximal regions of receptors (containing box domains) are sufficient for STAT activation suggest that JAK and STAT molecules could interact directly. The mechanisms by which STATs interact with JAKs are not fully understood, but seem to be independent of phosphotyrosine. Indeed, when Fujitani and al. studied the interaction between STAT5 with JAK1, they found that the region necessary for their association (amino acids 680–688) did not encompass STAT5's major phosphor‐acceptor site, namely Y694.^35^ Receptors using this ‘JAK‐direct’ activation of STATs are summarized in Figure 5.

### 3.1 IL‐6R

The IL‐6 family of cytokines encompasses IL‐6, IL‐11, IL‐27, LIF (leukaemia inhibitory factor), and a few other cytokines that have in common gp130 as one of their signalling chains. Gp130 is ubiquitously expressed and contains six tyrosine residues in its intracellular domain (Figure 5A). Upon IL‐6 stimulation, STAT1, STAT3 and STAT5 are activated. A direct interaction between JAK1 and STAT5 was demonstrated in 1997.^35^ Indeed, it has been shown that, upon stimulation of a gp130 mutant receptor in which all 6 tyrosine residues were mutated into phenylalanines, STAT5 was still activated to a similar extent as with the WT receptor. The same results were obtained with a receptor truncated right after the box 2 domain. Moreover, using the yeast two‐hybrid screening system, it was observed that STAT5 was able to interact directly with JAK proteins and therefore could be activated independently of tyrosine phosphorylation. The study showed that STAT5a and STAT‐5b could interact specifically with the JH2 domain of JAK1, JAK2 and JAK3. These results were confirmed in vitro using co‐immunoprecipitation and GST pull‐down experiments. In the same study, using a mutant lacking the YxxQ STAT3 activation motif, it was confirmed that STAT3 activation, unlike that of STAT5, was dependent on tyrosine phosphorylation of the gp130 intracellular domain and was independent of any contact with JH2 domains of JAK proteins. This was corroborated by another study that demonstrated the importance of specific phosphorylated tyrosine residues (Tyr767, Tyr814, Tyr905 and Tyr915) in the activation of STAT3. The last two of these tyrosine residues were also proven necessary for STAT1 phosphorylation.^37^ A direct interaction between JAK2 and STAT5 was also observed for the mouse oncostatin M receptor (OSM‐R), another member of gp130 family.^38^

To conclude, activation of STAT5 does not require the tyrosines of gp130 or OSM‐R, and can occur directly through JAK proteins, with which it has been shown to interact. This is not the case for STAT1 or STAT3.^35^

### 3.2 IL‐4R

IL‐4 signals through a bipartite receptor composed of IL‐4Rα and the common γ‐receptor chain (Figure 5B). IL‐4Rα can also form a complex with the IL‐13Rα chain to transduce IL‐4 signalling. Upon ligand binding, JAK1 and JAK3 (for γ‐chain) or JAK2 (for IL‐13Rα chain) are activated and induce mainly the activation of STAT6 but also of STAT5. IL‐4 functions are immune‐related, and triggering the IL‐4 signalling pathway leads to cell proliferation and differentiation. Like for gp130, several studies have shown an activation of STAT5 and STAT‐6 independently of the 6 tyrosines of the IL‐4Rα. Here again, a direct interaction between JAK1 and STAT5 has been described. By performing immunoprecipitations, Erhard et al^36^ observed a direct link between JAK1 and STAT5 even in unphosphorylated conditions. In other studies, when a truncated IL‐4Rα lacking all tyrosine residues but retaining the box 1 domain is stimulated with IL‐4, STAT5 and STAT‐6 activation is still observed and comparable to that obtained with the WT receptor.^39^, ^40^ This implies that the membrane‐proximal cytoplasmic portion of IL‐4Rα is sufficient for STAT activation. While this is coherent with previous data showing a direct interaction of STAT5 with JAK1,^36^ no such interaction has yet been detected for STAT6. It is also worth noting that activation of STAT5 and STAT‐6 is strictly dependent on JAK1, since when the box 1 domain required for JAK1 binding is truncated on both chains of the receptor (IL‐4Rα and γc), activation of these STATs is abolished.^39^, ^40^

### 3.3 EPO‐R

Signalling through the erythropoietin (EPO)‐receptor is crucial for the fate of erythroid progenitor cells. In the absence of EPO, EPO‐R is reported to exist in pre‐formed dimers at the cell surface. Binding of EPO changes the orientation of the two receptor subunits leading to activation of JAK2 and the subsequent phosphorylation of STAT1, STAT3 and STAT5 (Figure 5C).^41^, ^42^ Like the gp130 chain and IL‐4Rα, the EPO receptor has been shown to signal through classical and non‐classical JAK/STAT5 signalling pathways. Indeed, cells expressing an EPO‐R lacking its 8 tyrosines are still able to induce a low but detectable level of activation of STAT5. This indicates that STAT5 phosphorylation does not require direct binding to receptor tyrosines and could also be mediated by another region of the receptor, or even by another protein. JAK2 would be a good candidate for this since seven of its tyrosine residues show similarity with the YxxL motif identified in EPO‐R as crucial for STAT5 activation. Another argument in favour of this interaction is that the alternative, tyrosine‐independent mechanism of STAT5 activation requires a specific region within the first 95 membrane‐proximal amino acids of the receptor, which does not contain any tyrosines. Further studies are required to demonstrate that alternative STAT5 activation is mediated through direct STAT5‐JAK2 interaction. Of note, the alternative tyrosine‐independent pathway plays only a minor role in STAT5 phosphorylation compared to the classical phosphotyrosine‐dependent pathway. Indeed, a 100‐fold higher EPO concentration is needed to induce alternative STAT5 phosphorylation.^43^, ^44^

### 3.4 G‐CSF‐R

The biological effects of G‐CSF are mediated through homodimerization of G‐CSF receptor chains, which contain four tyrosine residues (Figure 2D). As mentioned in the first part of this review, upon G‐CSF stimulation, STAT1, STAT3 and STAT5 are activated. STAT3 activation can be mediated through tyrosine residues, but also through the C‐terminus of the receptor.^14^, ^15^ Moreover, a second tyrosine‐independent mechanism of STAT activation has been observed for the G‐CSF‐R, with STAT1 and STAT5 phosphorylation occurring even with a truncated G‐CSF‐R lacking all its tyrosines but keeping the conserved proline‐rich box 1 and 2 domains. In addition, the level of activation observed was similar to that by a full‐length receptor.^16^, ^45^, ^46^ Some studies have proposed that JAK proteins (JAK1 and JAK2) may recruit and phosphorylate STAT1 and STAT5 in the context of G‐CSF signalling, although none have demonstrated a direct interaction between JAKs and STATs.^23^, ^47^

### 3.5 GH‐R

Growth hormone (GH) is a cytokine that promotes cell division, regeneration and growth. It signals through a constitutive homodimeric receptor, comprising several intracellular tyrosine residues (Figure 5D). For this receptor, a mechanism of activation independent of tyrosines of the receptor has been identified for STAT1 and STAT3 but not for STAT5.^48^, ^49^ Indeed, several studies suggest that the membrane‐proximal domain of GH‐R is sufficient to induce a response mediated by STAT1 and STAT3.^50^, ^51^, ^52^, ^53^ Moreover, absence of GH‐R phosphorylation does not impact STAT3 phosphorylation.^54^ Accordingly, JAK2 is proposed to be a docking site for STAT1 and STAT3 in GH signalling, most probably via the STAT SH2 domains.^55^ No direct interaction between STAT1, STAT3 and JAK2 have, however, been shown in the context of GH‐R. GH‐R tyrosines are in contrast absolutely required for STAT5 phosphorylation. Their absence completely abolishes STAT5 activity in EMSA experiments^56^; moreover, multiple studies have demonstrated that tyrosines located in the C‐terminal part of GH‐R are essential for STAT5 signalling,^53^, ^54^, ^56^, ^57^, ^58^ although one suggests that two N‐terminal tyrosines are also involved.^58^ Of note, GH‐R from pig, rabbit, human and mouse has been used to study GH signalling, making the overall analysis difficult. Therefore, defining residues involved in signalling is complex.

### 3.6 IL‐7R

An IL‐7R (Figure 5E) lacking all tyrosine residues is still able to activate STAT5 signalling. This unconventional mechanism relies on an acidic region of the receptor located between K312 and L328.^59^ MEK1/2 seems to be implicated in this alternative STAT5 activation, as pSTAT5 signal is abolished when an inhibitor of MEK1/2 is used with a tyrosine‐less IL‐7R mutant model. Moreover, MEK1/2 and STAT5 co‐precipitate and MEK1/2 is able to directly phosphorylate STAT5 in vitro, supporting their interaction. This alternative mechanism seems to play a role in the differentiation of T αβ and T γδ lymphocytes: when a mutant receptor lacking the acidic region is introduced into T‐cell progenitors from *Il7Ra* ^−/−^ mice, it fails to restore αβ and γδ T‐cell maturation upon IL‐7 stimulation, while the wild‐type receptor does.^59^ Besides the noncanonical mechanism, Tyr449 of IL‐7Rα is also described to play important roles in IL‐7 signalling in vivo and in vitro.^60^, ^61^ Importantly, the T‐cell maturation is maximal when IL‐7R contains both its tyrosines and its acidic region, demonstrating that canonical and noncanonical mechanisms cooperate.^59^

### 3.7 IL‐2Rβ

IL‐2 signals through a heterotrimeric receptor composed of the IL‐2Rα chain, IL‐2Rβ chain and the γc chain (Figure 5F). Upon IL‐2 stimulation, JAK1 and JAK3 are activated and phosphorylate residues of the IL‐2Rβ and γc chains. Phosphorylated tyrosines of the receptor serve as docking sites for the recruitment of STAT1, STAT3 and STAT5.^62^, ^63^ The intracellular part of the IL‐2ß receptor can be divided into 3 domains: the proximal domain that contains no tyrosines but contains box 1 and 2, the acidic domain with four tyrosine residues, and the C‐ter containing the last two tyrosines.^62^ Using fusion proteins between GST and different parts of the receptor, the binding sites of STAT1, STAT3 and STAT5 were identified by the team of Bertoglio.^62^ For STAT5, Tyr510 seems to be the major docking site. As for STAT1 and STAT3, the activation mechanism was shown to be phosphotyrosine‐independent and instead dependent on the acidic region located between amino acids 335 and 364. First, STAT1 and STAT3 were found to bind the receptor even when the C‐ter was lacking. Second, STAT1 and STAT3 were still shown to associate, but drastically less, with a fusion protein containing only part of the acidic domain and in which all Tyr were mutated to Phe. This would imply that the tyrosine residues of the cytoplasmic domain are not required for the binding of these STATs. Nevertheless, it was not possible to reproduce the direct interaction between the receptor and STATs in a cellular context. It is also worth mentioning that the conclusions drawn here relied on binding assays only, with no data regarding signalling through a mutated/truncated IL‐2R.^62^ To Bertoglio team, the alternative mechanism does not appear to involve direct binding to JAK proteins since, to their knowledge, the acidic subdomain is not involved in the association with JAKs.^62^ However, another study demonstrated that aa 330 to 362 of the IL‐2Rβ does actually seem to be of importance in JAK3 binding,^64^ in favour of a direct link between STAT1, STAT3 and JAK3. Future studies would help to confirm this direct interaction or determine whether another adaptor protein is required in the cellular context.

### 3.8 Conclusions

The JAK‐direct mechanism appears to often be associated with STAT5 activation.^35^, ^36^ This, however, leads to a question: Why is this not a common mechanism used by all cytokine receptors signalling through the JAK/STAT5 pathway? For example, STAT5 activation through the IL‐22R requires tyrosine residues. Indeed, an IL‐22 receptor lacking all tyrosine residues is not able to induce STAT5 activation.^8^ This means that JAK‐direct interactions are context‐dependent and specific to certain receptors. Moreover, the importance of these ‘JAK‐direct’ mechanisms varies between receptors. Indeed, while the direct interactions between JAKs and STATs only account for a small part of the total STAT5 activation in EPO‐R, it is crucial for STAT5 signalling mediated by IL‐6. Interestingly, IL‐6 is not known to be a strong inducer of STAT5 but rather of STAT3, perhaps because all STAT5 activation passes through the alternative mechanism.

Only a few studies have been able to confirm a direct ‘physical’ interaction between JAK and STAT proteins.^35^, ^36^ Most of these interactions were demonstrated through GST pull down using cells overexpressing proteins of interest. There is thus clearly a lack of in vivo studies, or at least in vitro studies undertaken in more ‘physiological’ conditions, by truncation of the endogenous receptor via CRISPR/Cas9 for instance. This would help to define the functional role of these alternative mechanisms.

## 4 GENERAL CONCLUSIONS

In this review, we discuss two major alternative modes of STAT activation: one that is dependent on a tyrosine‐less region of the receptor, and the other involving a direct interaction between STATs and JAKs. Evidence shows that one or both alternative mechanisms add to the canonical activation of STATs by some but not all cytokine receptors. This begs the question: what could the advantages of such alternative mechanisms be?

A first advantage may be quantitative. Indeed, in general, the classical and alternative mechanisms cooperate to strongly induce STAT signalling. In IL‐22 signalling, for example, phosphotyrosine‐dependent and phosphotyrosine‐independent activations of STAT3 synergize. Even though the alternative activation mode seems to be more crucial to STAT3 activation, both are required to reach the full effect of IL‐22. In contrast, almost no data are available to determine whether alternative and canonical activations of STAT are qualitatively different in terms of regulation of gene expression. Based on our unpublished results, a quantitative advantage is more likely.

Second, the pre‐association of STAT to the receptor, demonstrated for IL‐22Rα, IFNAR or gp130 for example, may enable a more rapid response following cytokine binding, as STAT is more accessible than when it ‘swims’ freely in the cytoplasm. One can imagine that rapid signalling in response to IL‐22 or type I IFN is crucial to provide effective defence against bacterial or viral pathogens. Some groups have investigated the kinetics of ‘canonical’ and ‘noncanonical’ signalling. By time‐course studies in the context of G‐CSF‐R, it was shown that both tyrosine‐dependent and tyrosine‐independent mechanisms of STAT3 activation follow similar kinetics.^15^ As for the EPO‐R, the kinetics of activation and inactivation of STAT5 were the same even when the ‘JAK‐direct alternative mechanism’ of activation was used.^43^ These parameters have not yet been evaluated for other receptors.

Phosphotyrosine‐independent activation could also provide an advantage by prolonging STAT activation. Alternative signalling may escape negative feedback mechanisms that are receptor phosphotyrosine‐dependent. SOCS3 interferes with JAK/STAT signalling by binding to JAK and inhibiting its function. It can also bind receptor phosphotyrosines via its SH2 domain, competing with STAT for receptor binding.^65^ Therefore, phosphotyrosine‐independent mechanisms might partially escape the negative feedback by SOCS. Indeed, when SOCS3 and an IL‐22Rα lacking all tyrosines were co‐transfected into 293 cells, the alternative activation of STAT3 was significantly less sensitive to SOCS3‐negative feedback compared to canonical STAT3 activation induced by WT receptor (unpublished data from our laboratory). Therefore, the signalling dependent on the pre‐association of STATs with cytokine receptors does not seem to be inhibited by a retro‐feedback. The role of this negative feedback on JAK‐direct mechanisms has never been studied.

Of note, if STAT activation is sustained, it may lead to deleterious effects such as tumour development. Thus, in the future, it might be interesting to develop targeted therapies against these noncanonical pathways of STAT activation. Indeed, blocking alternative pathways while leaving the canonical ones intact might reduce JAK/STAT signalling while avoiding side effects due to complete loss of a cytokine pathway.

## CONFLICT OF INTEREST

The authors state no conflict of interest.

## AUTHOR CONTRIBUTIONS

**Lena Puigdevall:** Conceptualization (equal); Data curation (lead); Writing – original draft (lead); Writing – review & editing (equal). **Camille Michiels:** Conceptualization (equal); Data curation (lead); Writing – original draft (lead); Writing – review & editing (equal). **Clara Stewardson:** Writing – review & editing (equal). **Laure Dumoutier:** Conceptualization (lead); Data curation (equal); Funding acquisition (lead); Supervision (lead); Writing – review & editing (equal).

## References

[1] Haan C , Kreis S , Margue C , Behrmann I . Jaks and cytokine receptors–an intimate relationship. Biochem Pharmacol. 2006;72:1538‐1546. 10.1016/j.bcp.2006.04.013 16750817

[2] Baker SJ , Rane SG , Reddy EP . Hematopoietic cytokine receptor signaling. Oncogene. 2007;26:6724‐6737. 10.1038/sj.onc.1210757 17934481

[3] Yoshimura A , Ohkubo T , Kiguchi T , et al. A novel cytokine‐inducible gene CIS encodes an SH2‐containing protein that binds to tyrosine‐phosphorylated interleukin 3 and erythropoietin receptors. EMBO J. 1995;14:2816‐2826. 10.1002/j.1460-2075 7796808 PMC398400

[4] Khwaja A . The role of Janus kinases in haemopoiesis and haematological malignancy. Br J Haematol. 2006;134:366‐384. 10.1111/j.1365-2141.2006.06206.x 16822289

[5] Ungureanu D , Saharinen P , Junttila I , Hilton DJ , Silvennoinen O . Regulation of Jak2 through the ubiquitin‐proteasome pathway involves phosphorylation of Jak2 on Y1007 and interaction with SOCS‐1. Mol Cell Biol. 2002;22:3316‐3326. 10.1128/mcb.22.10.3316-3326 11971965 PMC133778

[6] Morris R , Kershaw NJ , Babon JJ . The molecular details of cytokine signaling via the JAK/STAT pathway. Protein Sci. 2018;27:1984‐2009. 10.1002/pro.3519 30267440 PMC6237706

[7] Demoulin JB , Uyttenhove C , Van Roost E , et al. A single tyrosine of the interleukin‐9 (IL‐9) receptor is required for STAT activation, antiapoptotic activity, and growth regulation by IL‐9. Mol Cell Biol. 1996;16:4710‐4716. 10.1128/mcb.16.9.4710 8756628 PMC231471

[8] Dumoutier L , de Meester C , Tavernier J , Renauld JC . New activation modus of STAT3: a tyrosine‐less region of the interleukin‐22 receptor recruits STAT3 by interacting with its coiled‐coil domain. J Biol Chem. 2009;284:26377‐26384. 10.1074/jbc.M109.007955 19632985 PMC2785325

[9] La Sala G , Michiels C , Kükenshöner T , et al. Selective inhibition of STAT3 signaling using monobodies targeting the coiled‐coil and N‐terminal domains. Nat Commun. 2020;11:4115. 10.1038/s41467-020-17920-z 32807795 PMC7431413

[10] Michiels C , Puigdevall L , Cochez P , et al. A targetable, noncanonical signal transducer and activator of transcription 3 activation induced by the Y‐less region of IL‐22 receptor orchestrates imiquimod‐induced psoriasis‐like dermatitis in mice. J Invest Dermatol. 2021;141(11):2668‐2678.e6. 10.1016/j.jid.2021.04.016 33992648

[11] Dumoutier L , Tounsi A , Michiels T , Sommereyns C , Kotenko SV , Renauld JC . Role of the interleukin (IL)‐28 receptor tyrosine residues for antiviral and antiproliferative activity of IL‐29/interferon‐lambda 1: similarities with type I interferon signaling. J Biol Chem. 2004;279:32269‐32274. 10.1074/jbc.M404789200 15166220

[12] Floss DM , Schroder J , Franke M , Scheller J . Insights into IL‐23 biology: from structure to function. Cytokine Growth Factor Rev. 2015;26:569‐578. 10.1016/j.cytogfr.2015.07.005 26195433

[13] Floss DM , Mrotzek S , Klöcker T , et al. Identification of canonical tyrosine‐dependent and non‐canonical tyrosine‐independent STAT3 activation sites in the intracellular domain of the interleukin 23 receptor. J Biol Chem. 2013;288:19386‐19400. 10.1074/jbc.M112.432153 23673666 PMC3707643

[14] Nicholson SE , Starr R , Novak U , Hilton DJ , Layton JE . Tyrosine residues in the granulocyte colony‐stimulating factor (G‐CSF) receptor mediate G‐CSF‐induced differentiation of murine myeloid leukemic (M1) cells. J Biol Chem. 1996;271:26947‐26953. 10.1074/jbc.271.43.26947 8900180

[15] Ward AC , Hermans MHA , Smith L , et al. Tyrosine‐dependent and ‐independent mechanisms of STAT3 activation by the human granulocyte colony‐stimulating factor (G‐CSF) receptor are differentially utilized depending on G‐CSF concentration. Blood. 1999;93:113‐124. 10.1182/blood.V93.1.113 9864153

[16] Tian SS , Tapley P , Sincich C , Stein RB , Rosen J , Lamb P . Multiple signaling pathways induced by granulocyte colony‐stimulating factor involving activation of JAKs, STAT5, and/or STAT3 are required for regulation of three distinct classes of immediate early genes. Blood. 1996;88:4435‐4444. 10.1182/blood.V88.12.4435 8977235

[17] Mehta HM , Futami M , Glaubach T , et al. Alternatively spliced, truncated GCSF receptor promotes leukemogenic properties and sensitivity to JAK inhibition. Leukemia. 2014;28:1041‐1051. 10.1038/leu.2013.321 24170028 PMC5875430

[18] Liu F , Kunter G , Krem MM , et al. Csf3r mutations in mice confer a strong clonal HSC advantage via activation of Stat5. J Clin Invest. 2008;118:946‐955. 10.1172/JCI32704 18292815 PMC2248325

[19] Qiu Y , Zhang Y , Hu N , Dong F . A truncated granulocyte colony‐stimulating factor receptor (G‐CSFR) inhibits apoptosis induced by neutrophil elastase G185R mutant: implication for understanding CSF3R gene mutations in severe congenital neutropenia. J Biol Chem. 2017;292:3496‐3505. 10.1074/jbc.M116.755157 28073911 PMC5336180

[20] Touw IP . Game of clones: the genomic evolution of severe congenital neutropenia. Hematology Am Soc Hematol Educ Program. 2015;1–7:2015. 10.1182/asheducation-2015.1.1 26637693

[21] Zhao W , Lee C , Piganis R , et al. A conserved IFN‐alpha receptor tyrosine motif directs the biological response to type I IFNs. J Immunol. 2008;180:5483‐5489. 10.4049/jimmunol.180.8.5483 18390731

[22] Li X , Leung S , Kerr IM , Stark GR . Functional subdomains of STAT2 required for preassociation with the alpha interferon receptor and for signaling. Mol Cell Biol. 1997;17:2048‐2056. 10.1128/mcb.17.4.2048 9121453 PMC232052

[23] Nadeau OW , Domanski P , Usacheva A , et al. The proximal tyrosines of the cytoplasmic domain of the beta chain of the type I interferon receptor are essential for signal transducer and activator of transcription (Stat) 2 activation. Evidence that two Stat2 sites are required to reach a threshold of interferon alpha‐induced Stat2 tyrosine phosphorylation that allows normal formation of interferon‐stimulated gene factor 3. J Biol Chem. 1999;274:4045‐4052. 10.1074/jbc.274.7.4045 9933596

[24] Russell‐Harde D , Wagner TC , Rani MRS , et al. Role of the intracellular domain of the human type I interferon receptor 2 chain (IFNAR2c) in interferon signaling. Expression of IFNAR2c truncation mutants in U5A cells. J Biol Chem. 2000;275:23981‐23985. 10.1074/jbc.M002518200 10825167

[25] Wagner TC , Velichko S , Vogel D , et al. Interferon signaling is dependent on specific tyrosines located within the intracellular domain of IFNAR2c. Expression of IFNAR2c tyrosine mutants in U5A cells. J Biol Chem. 2002;277:1493‐1499. 10.1074/jbc.M108928200 11682488

[26] Abramovich C , Shulman LM , Ratovitski E , et al. Differential tyrosine phosphorylation of the IFNAR chain of the type I interferon receptor and of an associated surface protein in response to IFN‐alpha and IFN‐beta. EMBO J. 1994;13:5871‐5877. 10.1002/j.1460-2075 7813427 PMC395562

[27] Qureshi SA , Leung S , Kerr IM , Stark GR , Darnell JE Jr . Function of Stat2 protein in transcriptional activation by alpha interferon. Mol Cell Biol. 1996;16:288‐293. 10.1128/mcb.16.1.288 8524306 PMC231002

[28] Nguyen VP , Saleh AZ , Arch AE , et al. Stat2 binding to the interferon‐alpha receptor 2 subunit is not required for interferon‐alpha signaling. J Biol Chem. 2002;277:9713‐9721. 10.1074/jbc.M111161200 11786546

[29] Ferrao R , Lupardus PJ . The Janus kinase (JAK) FERM and SH2 domains: bringing specificity to JAK‐receptor interactions. Front Endocrinol (Lausanne). 2017;8:71. 10.3389/fendo.2017.00071 28458652 PMC5394478

[30] Jaber Chehayeb R , Boggon TJ . SH2 domain binding: diverse FLVRs of partnership. Front Endocrinol (Lausanne). 2020;11:575220. 10.3389/fendo.2020.575220 33042028 PMC7530234

[31] Leung S , Qureshi SA , Kerr IM , Darnell JE Jr , Stark GR . Role of STAT2 in the alpha interferon signaling pathway. Mol Cell Biol. 1995;15:1312‐1317. 10.1128/mcb.15.3.1312 7532278 PMC230354

[32] Velichko S , Wagner TC , Turkson J , Jove R , Croze E . STAT3 activation by type I interferons is dependent on specific tyrosines located in the cytoplasmic domain of interferon receptor chain 2c. Activation of multiple STATS proceeds through the redundant usage of two tyrosine residues. J Biol Chem. 2002;277:35635‐35641. 10.1074/jbc.M204578200 12105218

[33] Li WX . Canonical and non‐canonical JAK‐STAT signaling. Trends Cell Biol. 2008;18:545‐551. 10.1016/j.tcb.2008.08.008 18848449 PMC3082280

[34] Dutta P , Zhang L , Zhang H , et al. Unphosphorylated STAT3 in heterochromatin formation and tumor suppression in lung cancer. BMC Cancer. 2020;20:145. 10.1186/s12885-020-6649-2 32087696 PMC7036253

[35] Fujitani Y , Hibi M , Fukada T , et al. An alternative pathway for STAT activation that is mediated by the direct interaction between JAK and STAT. Oncogene. 1997;14:751‐761. 10.1038/sj.onc.1200907 9047382

[36] Erhardt I , Lischke A , Sebald W , Friedrich K . Constitutive association of JAK1 and STAT5 in pro‐B cells is dissolved by interleukin‐4‐induced tyrosine phosphorylation of both proteins. FEBS Lett. 1998;439:71‐74. 10.1016/j.imlet.2007.01.008 9849880

[37] Gerhartz C , Heesel B , Sasse J , et al. Differential activation of acute phase response factor/STAT3 and STAT1 via the cytoplasmic domain of the interleukin 6 signal transducer gp130. I. Definition of a novel phosphotyrosine motif mediating STAT1 activation. J Biol Chem. 1996;271:12991‐12998. 10.1074/jbc.271.22.12991 8662591

[38] Hintzen C , Evers C , Lippok BE , et al. Box 2 region of the oncostatin M receptor determines specificity for recruitment of Janus kinases and STAT5 activation. J Biol Chem. 2008;283:19465‐19477. 10.1074/jbc.M710157200 18430728

[39] Friedrich K , Kammer W , Erhardt I , Brändlein S , Sebald W , Moriggl R . Activation of STAT5 by IL‐4 relies on Janus kinase function but not on receptor tyrosine phosphorylation, and can contribute to both cell proliferation and gene regulation. Int Immunol. 1999;11:1283‐1294. 10.1093/intimm/11.8.1283 10421786

[40] Moriggl R , Erhardt I , Kammer W , et al. Activation of STAT6 is not dependent on phosphotyrosine‐mediated docking to the interleukin‐4 receptor and can be blocked by dominant‐negative mutants of both receptor subunits. Eur J Biochem. 1998;251:25‐35. 10.1046/j.1432-1327.1998.2510025.x 9492265

[41] Kirito K , Nakajima K , Watanabe T , et al. Identification of the human erythropoietin receptor region required for Stat1 and Stat3 activation. Blood. 2002;99:102‐110. 10.1182/blood.v99.1.102 11756159

[42] Lu X , Gross AW , Lodish HF . Active conformation of the erythropoietin receptor: random and cysteine‐scanning mutagenesis of the extracellular juxtamembrane and transmembrane domains. J Biol Chem. 2006;281:7002‐7011. 10.1074/jbc.M512638200 16414957

[43] Klingmuller U , Bergelson S , Hsiao JG , Lodish HF . Multiple tyrosine residues in the cytosolic domain of the erythropoietin receptor promote activation of STAT5. Proc Natl Acad Sci USA. 1996;93:8324‐8328. 10.1073/pnas.93.16.8324 8710869 PMC38669

[44] Damen JE , Wakao H , Miyajima A , et al. Tyrosine 343 in the erythropoietin receptor positively regulates erythropoietin‐induced cell proliferation and Stat5 activation. EMBO J. 1995;14:5557‐5568. 8521813 10.1002/j.1460-2075.1995.tb00243.x PMC394670

[45] de Koning JP , Dong F , Smith L , et al. The membrane‐distal cytoplasmic region of human granulocyte colony‐stimulating factor receptor is required for STAT3 but not STAT1 homodimer formation. Blood. 1996;87:1335‐1342. 10.1182/blood.V87.4.1335 8608222

[46] Dong F , Liu X , de Koning JP , et al. Stimulation of Stat5 by granulocyte colony‐stimulating factor (G‐CSF) is modulated by two distinct cytoplasmic regions of the G‐CSF receptor. J Immunol. 1998;161:6503‐6509. 9862674

[47] Liongue C , Ward AC . Granulocyte colony‐stimulating factor receptor mutations in myeloid malignancy. Front Oncol. 2014;4:93. 10.3389/fonc.2014.00093 24822171 PMC4013473

[48] Brooks AJ , Waters MJ . The growth hormone receptor: mechanism of activation and clinical implications. Nat Rev Endocrinol. 2010;6:515‐525. 10.1038/nrendo.2010.123 20664532

[49] Herrington J , Smit LS , Schwartz J , Carter‐Su C . The role of STAT proteins in growth hormone signaling. Oncogene. 2000;19:2585‐2597. 10.1038/sj.onc.1203526 10851057

[50] Sotiropoulos A , Moutoussamy S , Binart N , Kelly PA , Finidori J . The membrane proximal region of the cytoplasmic domain of the growth hormone receptor is involved in the activation of Stat 3. FEBS Lett. 1995;369:169‐172. 10.1016/0014-5793(95)00734-q 7649252

[51] Wang YD , Wong K , Wood WI . Intracellular tyrosine residues of the human growth hormone receptor are not required for the signaling of proliferation or Jak‐STAT activation. J Biol Chem. 1995;270:7021‐7024. 10.1074/jbc.270.13.7021 7535764

[52] Hackett RH , Wang YD , Larner AC . Mapping of the cytoplasmic domain of the human growth hormone receptor required for the activation of Jak2 and Stat proteins. J Biol Chem. 1995;270:21326‐21330. 10.1074/jbc.270.36.21326 7673169

[53] Yi W , Kim SO , Jiang J , et al. Growth hormone receptor cytoplasmic domain differentially promotes tyrosine phosphorylation of signal transducers and activators of transcription 5b and 3 by activated JAK2 kinase. Mol Endocrinol. 1996;10:1425‐1443. 10.1210/mend.10.11.8923468 8923468

[54] Sotiropoulos A , Moutoussamy S , Renaudie F , et al. Differential activation of Stat3 and Stat5 by distinct regions of the growth hormone receptor. Mol Endocrinol. 1996;10:998‐1009. 10.1210/mend.10.8.8843416 8843416

[55] Murray PJ . The JAK‐STAT signaling pathway: input and output integration. J Immunol. 2007;178:2623‐2629. 10.4049/jimmunol.178.5.2623 17312100

[56] Hansen LH , Wang X , Kopchick JJ , et al. Identification of tyrosine residues in the intracellular domain of the growth hormone receptor required for transcriptional signaling and Stat5 activation. J Biol Chem. 1996;271:12669‐12673. 10.1074/jbc.271.21.12669 8647880

[57] Wang X , Darus CJ , Xu BC , Kopchick JJ . Identification of growth hormone receptor (GHR) tyrosine residues required for GHR phosphorylation and JAK2 and STAT5 activation. Mol Endocrinol. 1996;10:1249‐1260. 10.1210/mend.10.10.9121492 9121492

[58] Smit LS , Meyer DJ , Billestrup N , et al. The role of the growth hormone (GH) receptor and JAK1 and JAK2 kinases in the activation of Stats 1, 3, and 5 by GH. Mol Endocrinol. 1996;10:519‐533. 10.1210/mend.10.5.8732683 8732683

[59] Maki K , Ikuta K . MEK1/2 induces STAT5‐mediated germline transcription of the TCRgamma locus in response to IL‐7R signaling. J Immunol. 2008;181:494‐502. 10.4049/jimmunol.181.1.494 18566415

[60] Jiang Q , Li WQ , Hofmeister RR , et al. Distinct regions of the interleukin‐7 receptor regulate different Bcl2 family members. Mol Cell Biol. 2004;24:6501‐6513. 10.1128/MCB.24.14.6501-6513.2004 15226449 PMC434255

[61] Osborne LC , Dhanji S , Snow JW , et al. Impaired CD8 T cell memory and CD4 T cell primary responses in IL‐7R alpha mutant mice. J Exp Med. 2007;204:619‐631. 10.1084/jem.20061871 17325202 PMC2137912

[62] Delespine‐Carmagnat M , Bouvier G , Bertoglio J . Association of STAT1, STAT3 and STAT5 proteins with the IL‐2 receptor involves different subdomains of the IL‐2 receptor beta chain. Eur J Immunol. 2000;30:59‐68. 10.1002/1521-4141(200001)30:1<59:AID-IMMU59>3.0.CO;2-1 10602027

[63] Fujii H , Nakagawa Y , Schindler U , et al. Activation of Stat5 by interleukin 2 requires a carboxyl‐terminal region of the interleukin 2 receptor beta chain but is not essential for the proliferative signal transmission. Proc Natl Acad Sci USA. 1995;92:5482‐5486. 10.1073/pnas.92.12.5482 7777534 PMC41719

[64] Zhu MH , Berry JA , Russell SM , Leonard WJ . Delineation of the regions of interleukin‐2 (IL‐2) receptor beta chain important for association of Jak1 and Jak3. Jak1‐independent functional recruitment of Jak3 to Il‐2Rbeta. J Biol Chem. 1998;273:10719‐10725. 10.1074/jbc.273.17.10719 9553136

[65] Ilangumaran S , Ramanathan S , Rottapel R . Regulation of the immune system by SOCS family adaptor proteins. Semin Immunol. 2004;16:351‐365. 10.1016/j.smim.2004.08.015 15541651

